# Molecular and Low-Resolution Structural Characterization of the Na^+^-Translocating Glutaconyl-CoA Decarboxylase From *Clostridium symbiosum*

**DOI:** 10.3389/fmicb.2020.00480

**Published:** 2020-03-31

**Authors:** Stella Vitt, Simone Prinz, Nils Hellwig, Nina Morgner, Ulrich Ermler, Wolfgang Buckel

**Affiliations:** ^1^Department of Molecular Membrane Biology, Max Planck Institute of Biophysics, Frankfurt, Germany; ^2^Faculty of Biology, Philipps-Universität Marburg, Marburg, Germany; ^3^Department of Structural Biology, Max Planck Institute of Biophysics, Frankfurt, Germany; ^4^Institute of Physical and Theoretical Chemistry, Goethe University Frankfurt, Frankfurt, Germany

**Keywords:** anaerobic energy metabolism, glutaconyl-CoA decarboxylase, ion translocation, biotin, negative-stain electron microscopy, LILBID-MS

## Abstract

Some anaerobic bacteria use biotin-dependent Na^+^-translocating decarboxylases (Bdc) of β-keto acids or their thioester analogs as key enzymes in their energy metabolism. Glutaconyl-CoA decarboxylase (Gcd), a member of this protein family, drives the endergonic translocation of Na^+^ across the membrane with the exergonic decarboxylation of glutaconyl-CoA (Δ*G*^0^’ ≈−30 kJ/mol) to crotonyl-CoA. Here, we report on the molecular characterization of Gcd from *Clostridium symbiosum* based on native PAGE, size exclusion chromatography (SEC) and laser-induced liquid bead ion desorption mass spectrometry (LILBID-MS). The obtained molecular mass of ca. 400 kDa fits to the DNA sequence-derived mass of 379 kDa with a subunit composition of 4 GcdA (65 kDa), 2 GcdB (35 kDa), GcdC1 (15 kDa), GcdC2 (14 kDa), and 2 GcdD (10 kDa). Low-resolution structural information was achieved from preliminary electron microscopic (EM) measurements, which resulted in a 3D reconstruction model based on negative-stained particles. The Gcd structure is built up of a membrane-spanning base primarily composed of the GcdB dimer and a solvent-exposed head with the GcdA tetramer as major component. Both globular parts are bridged by a linker presumably built up of segments of GcdC1, GcdC2 and the 2 GcdDs. The structure of the highly mobile Gcd complex represents a template for the global architecture of the Bdc family.

## Introduction

The Earth’s biogeochemical carbon cycle involves the microbial decomposition of large quantities of chemically diverse organic compounds primarily derived from carbohydrates, lipids and proteins. While aerobic organisms predominantly apply the respiratory chain for ATP synthesis, anaerobically living microorganisms mainly use substrate-level phosphorylation for this purpose. In addition, anaerobic bacteria developed special electrochemical ion-gradient forming enzyme machineries for commonly occurring exergonic reactions (or reaction types) in their metabolisms to conserve smaller energy increments. An example is the family of biotin-dependent Na^+^-translocating decarboxylases (Bdc) that use for ion pumping the exergonic decarboxylation of β-keto acids or β-carboxy thioesters and their vinylogues (Δ*G*^0^′≈ ca. −30 kJ/mol), abundant metabolites in anaerobic habitats (Buckel, 2001). The Bdc family consists of three biochemically studied subfamilies with different substrate specificities and probably many unexplored representatives in the available genomes. Glutaconyl-CoA decarboxylase (Gcd) has been discovered in the glutamate fermenters *Acidaminococcus fermentans* (Buckel and Semmler, 1982), *Peptostreptococcus asaccharolyticus* (Buckel and Semmler, 1983), *Clostridium symbiosum* (Buckel and Semmler, 1983), and *Fusobacterium nucleatum* (Beatrix et al., 1990) as well as in the benzoate oxidizer/synthesizer *Syntrophus aciditrophicus* (Mouttaki et al., 2007; Kim, 2011). Oxaloacetate decarboxylase (Oad) is mainly found in citrate fermenting enterobacteria (*Klebsiella pneumoniae*, *Salmonella typhimurium*, and *Vibrio cholerae*) (Dimroth, 1980, 1981; Dimroth and Thomer, 1983; Laussermair et al., 1989), whereas (*S*)-methylmalonyl-CoA decarboxylase (Mmd) is present in lactate and succinate fermenting anaerobic bacteria (*Veillonella parvula*, formerly called *Veillonella alcalescens*, and *Propionigenium modestum*) (Hilpert and Dimroth, 1983; Huder and Dimroth, 1993; Bott et al., 1997). Na^+^-translocation coupled to the decarboxylation of the corresponding substrates was demonstrated for Oad (Dimroth, 1980, 1981), Mmd (Hilpert and Dimroth, 1983) and Gcd (Buckel and Semmler, 1982, 1983) with inverted membrane vesicles or purified proteins after incorporation into artificial liposomes.

Oxaloacetate decarboxylase from *K. pneumoniae* is composed of three different subunits, OadA (64 kDa), OadB (45 kDa) and OadD (9 kDa). The N-terminal carboxytransferase domain (∼54 kDa) of the homodimeric OadA catalyzes the transfer of the β-carboxylate of oxaloacetate to biotin covalently attached to a lysine of the C-terminal biotin carrier domain (∼10 kDa) (Schwarz et al., 1988). OadB, an extremely hydrophobic integral membrane protein (11 putative transmembrane helices), catalyzes the decarboxylation of N^1^-carboxybiotin coupled to Na^+^ translocation. OadD contains one N-terminal transmembrane helix, a potentially flexible alanine/proline-rich (AP) linker and an C-terminal segment that binds to an about 30 amino acid long peptide of OadA (Dahinden et al., 2005). Hence, OadD connects OadA with OadB (Studer et al., 2007). Site-directed mutagenesis data identified OadB as the site of biotin decarboxylation and Na^+^-transport (Di Berardino and Dimroth, 1996).

Gcds from *A. fermentans* and *F. nucleatum* consist of four subunits GcdA, GcdB, GcdC and GcdD; the *C. symbiosum* enzyme harbors instead of one GcdC two slightly different subunits, GcdC1 and GcdC2. The hydrophilic subunits GcdA from *A. fermentans* and *C. symbiosum* (65 kDa each) catalyze the carboxyl transfer from glutaconyl-CoA onto biotin yielding crotonyl-CoA and N^1^-carboxybiotin (Eq. 1) (Buckel and Liedtke, 1986; Bendrat and Buckel, 1993). Biotin is covalently bound to the conserved lysine in the MKM sequence of GcdC from *A. fermentans* (14 kDa) (Buckel and Liedtke, 1986; Bendrat and Buckel, 1993; Braune et al., 1999) or to GcdC1 and C2 from *C. symbiosum* (15 and 14 kDa) (Kress et al., 2009). The membrane-soanning subunit GcdB (39 kDa, 11 putative trans membrane helices) catalyzes the decarboxylation N^1^-carboxybiotin coupled to Na^+^ translocation (Eq. 2).

(1) $$\mathrm{GcdA}:\mathrm{Glutaconyl}\,\text{-}\,\mathrm{CoA}+\mathrm{Biotin}\,\text{-}\,\mathrm{GcdC}⇌\text{Crotonyl-CoA}+{\text{N}}^{1}\,{\text{-Carboxybiotin-GcdC}}^{-};$$

(2) $$\text{GcdB}:N^{1}\,{\text{-Carboxybiotin-GcdC}}^{-}+{\text{H}}^{+}⇌\text{Biotin-GcdC}+{\text{CO}}_{2};$$

According to primary structure analysis, GcdC is composed of an N-terminal region serving as anchor to the residual protein, a flexible AP region and a C-terminal region containing the biotin-binding site. In GcdC1/GcdC2 of *C. symbiosum*, the N-terminal region consists of 30 amino acids, the AP region of 48/39 amino acids (thereof 26/22 alanine and 14/13 proline) and the C-terminal region of 71 and 70 amino acids. The small subunit GcdD (10 kDa) is built up of one N-terminal transmembrane α-helix (13–37 in *C. symbiosum*), an AP-rich region (51–60) and a C-terminal region (61–95) which may help to stabilize the whole Gcd complex (Buckel and Liedtke, 1986; Braune et al., 1999).

A comprehensive structural and mechanistic understanding of a complete Bdc complex requires detailed structural data. X-ray structures are determined for recombinant *K. pneumoniae* OadA (Studer et al., 2007) as well as for *A. fermentans* and *C. symbiosum* GcdAs (Wendt et al., 2003; Kress et al., 2009); the latter are arranged as homotetramers composed of a dimer of dimers. Proposed symmetrical and asymmetrical models have in common that GcdA sits in the cytoplasm directly above the membrane-spanning GcdB and the flexible GcdC, loaded with carboxylated biotin, swings from the GcdA to GcdB and returns back to GcdA after decarboxylation. The models differ in the arrangements of the putative CO_2_ and Na^+^ channels. The GcdD subunits were not considered in both models. As all attempts to solve a complete Bdc structure failed, only little information about the quaternary structures exists. For the Oad complex, the stoichiometric ratio for OadA, OadB, and OadD was reported to be 1:1:1 (Granjon et al., 2010); the stoichiometric compositions for Gcd are unknown. In this report, we prepared Gcd from *C. symbiosum* (Kress et al., 2009) and analyzed its size and subunit composition by SDS and native PAGE, laser induced liquid bead ion desorption-mass spectrometry (LILBID-MS) and size exclusion chromatography (SEC). Based on negative-stain single particle electron microscopy (EM), we were able to provide a first view of its global architecture.

## Materials and Methods

### Strains Growth Media

*C. symbiosum* (DSM 934), *P. asaccharolyticus* (DSM 20464), *A. fermentans* (DSM 20731), and *F. nucleatum* (DSM 15643) were obtained from the Deutsche Sammlung von Mikroorganismen (DSMZ, Braunschweig, Germany). The glutamate fermenting bacteria were grown anaerobically at 37°C on glutamate/yeast extract media in 5-L batch cultures supplemented with biotin (0.2 mg/L) and in the case of *F. nucleatum* additionally with 1% tryptone (Beatrix et al., 1990). Cells were harvested in the late exponential or early stationary phase under aerobic conditions and stored at −80°C.

### Protein Purification

For purification of Gcd from *C. symbiosum*, *P. asaccharolyticus*, *A. fermentans*, and *F. nucleatum* (Buckel and Semmler, 1983; Beatrix et al., 1990), 10 g wet cells were suspended in 25 ml lysis buffer (50 mM phosphate, pH 7.0), and passed four times through a French pressure cell at 125 MPa. Cell debris were removed by centrifugation at 10,000 × *g* and 4°C for 20 min. Membranes were subsequently collected by centrifugation at 190,000 × *g* and 4°C for 1.5 h and washed with lysis buffer. Gcd was solubilized with 2% 2-dodecyl β-D-maltoside (DDM) and high salt concentrations in buffer A (50 mM phosphate pH 7.0, 400 mM NaCl, 0.05% DDM) and low protein concentrations (0.5 mg/ml) at room temperature for 1 h. Membrane particles were removed at 190,000 × *g* and 4°C for 30 min. The solubilized enzymes were immediately loaded under low flow on a 5-ml monomeric avidin agarose column (Pierce, Thermo Scientific, Dreieich, Germany). Gcd was eluted in a biotin step gradient (0–2 mM biotin) in buffer A. The quality of the purified Gcd was examined by SDS-PAGE and enzymatic activity measurements after standard protocols. In these measurements, the formation of crotonyl-CoA, the product of the decarboxylation of glutaconyl-CoA (Eq. 1), was followed spectrophotometrically at 340 nm using a coupled enzymatic assay as described (Buckel and Semmler, 1982; Buckel and Liedtke, 1986).

### Gradient Fixation (GraFix)

Linear gradients were prepared according to standard protocols (Kastner et al., 2008) with some variations. The lighter buffer contained 50 mM phosphate, pH 7.0, 400 mM NaCl, and 10% (v/v) glycerol and the bottom buffer 50 mM phosphate, pH 7.0, 400 mM NaCl, 30% (v/v) glycerol, and 0.15% glutaraldehyde. Buffers were prepared by passing a filter of 0.2 μm pore size. After incubating for one hour at 4°C, a 50 pmol sample was loaded in a volume of 200 μl onto the prepared 4 ml GraFix gradient. Ultracentrifugation was carried out at 4°C in swing-out rotors (SW60 rotors, Beckmann) for 17 h with a speed of 50,000 rpm. After centrifugation the gradient was fractionated from bottom to top in 200 μl samples. Afterward fractions containing Gcd were pooled and loaded onto a Superose 6 10/300 SEC column equilibrated with 50 mM Tris–HCl pH 7.0, 200 mM NaCl, 0.05% DDM and eluted with a flow rate of 0.3 ml/min.

### Native PAGE

Clear native PAGE 4–16% Bis-Tris gels (Invitrogen, Thermo Fisher, Waltham, Massachusetts, United States) were run at 4°C with a cathode buffer supplemented with 0.05% DDM. Anode buffer, sample buffer and running conditions were performed as specified in the manual.

## Laser-Induced Liquid Bead Ion Desorption Mass Spectrometry (LILBID-MS)

Protein droplets of 50 μm diameter were prepared with a frequency of 10 Hz at 100 mbar using a piezo-driven droplet generator (MD-K-130 from Microdrop Technologies GmbH, Norderstedt, Germany). The generated droplets were transferred to high vacuum and irradiated by an Nd:YAG IR laser at 10 Hz with a pulse length of 6 ns and a maximum energy of 23 mJ. Its wavelength was set to 2.940 ± 0.005 μm by a LiNbO_3_ optical parametric oscillator.

The solvated ions were analyzed in a homebuilt time-of-flight setup including a reflectron, operating at 10^–6^ mbar, a Wiley-McLaren type accelerator and a Daly type based detector. Settings were used as described in detail earlier (Peetz et al., 2019). Spectra were processed by using the software *Mass*ign (Morgner and Robinson, 2012) based on LabVIEW. Spectra deconvolution was performed using UniDec (Marty et al., 2015).

### Negative-Stain Electron Microscopy

The Gcd complex was negatively stained with 2% (w/v) uranyl formate. A 3 μl protein sample was pipetted onto freshly glow-discharged carbon-coated copper grids. Electron micrographs were collected using a Tecnai^TM^ Spirit 12G transmission electron microscope (ThermoFisher–formerly FEI) operating at 120 kV at a nominal magnification of 42,000× and a calibrated pixel size of 2.68 Å (nominal defocus – 1.50 μm).

A set of 800 micrographs were collected on a GATAN 4096 × 4096 CCD detector by automated data collection with the Leginon software package (Suloway et al., 2005). Image processing, automatic particle picking, 2D classification and *ab initio* 3D map generation were performed with cisTEM (Grant et al., 2018) and RELION-3.0 (Zivanov et al., 2018).

## Results

### Purification of Several Gcd Complexes

For finding the optimal Gcd candidate for molecular and structural studies, *C. symbiosum, A. fermentans, F. nucleatum*, and *P. asaccharolyticus* were cultivated. Their Gcds were subjected to the standard preparation protocol with DDM for solubilization and monomeric avidin agarose affinity chromatography at high NaCl concentrations for purification. Subsequent SEC analysis indicated that the Gcd complexes from *F. nucleatum* and *P. asaccharolyticus* aggregated during purification as seen in the elution profile of affinity chromatography and subsequent SEC (Supplementary Figures S1A,B). At first glance the corresponding SEC profiles of Gcd from *A. fermentans* appeared more encouraging (Supplementary Figure S1C). Subsequent negative-stain EM analysis, however, revealed a crowded picture of aggregates and dissociated components, mainly consisting of the solvent-exposed head and the membrane-spanning base of Gcd (Supplementary Figure S1D). Further optimization by varying the pH, the buffer composition, the type and concentration of salt, and the detergent did not markedly improve the homogeneity of the protein.

**FIGURE 1:**
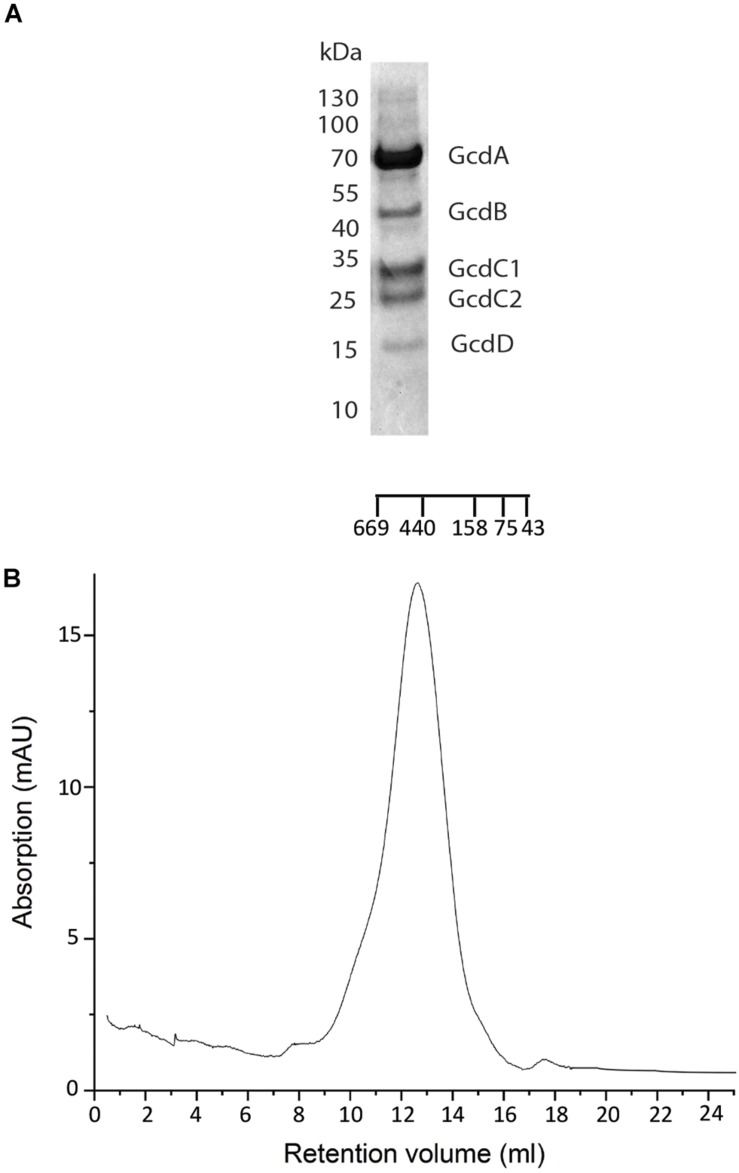
Purification of Gcd from *C. symbiosum*. **(A)** SDS-PAGE analysis on Gcd after the monomeric avidin agarose affinity chromatography. The compositions of the bands were identified by MALDI-TOF MS. **(B)** SEC profile of chemically fixed and unconcentrated Gcd. The SEC was performed with Tris–HCl, pH 7.0, 100 mM NaCl, 0,05% DDM.

Preparation of Gcd from *C. symbiosum* appeared to be most promising. When using buffer A, the SEC profile indicated aggregates, a monodisperse complex and small amounts of smaller fragments (Supplementary Figure S1E). In addition, the five predicted subunits GcdA, GcdB, GcdC1, and GcdC2 and GcdD could be identified by SDS-PAGE (Kress et al., 2009) and by MALDI-TOF (Figure 1A) and the yield of purified 3 mg Gcd per 10 g wet cells was sufficient for further studies. Purified Gcd was enzymatically active with respect to crotonyl-CoA formation (Buckel and Semmler, 1982; Buckel and Liedtke, 1986). The first negative-stain EM images showed separate and intact complex particles next to larger aggregates, but only minor amounts of small fragments. For increasing the ratio of monodisperse to aggregated Gcd complexes, we performed numerous optimization trials including twin-strep-tag chromatography (with two strep-tag motifs), other detergents such as lauryl maltose neopentyl glycol (LMNG), decyl glucose neopentyl glycol (DGNG), glyco-diosgenin (GDN), digitonin, and Triton X-100 for solubilization and purification (among others) or the diisobutylene maleic acid co-polymer (DIBMA), styrene-maleic acid (SMA) co-polymers, and amphipols as well as diverse pH and salt variations. However, negative-stain EM images and SEC profiles showed no significant improvements.

The only successful attempt to increase the fraction of intact and separated Gcd complexes was achieved with the GraFix method, by which after affinity chromatography the protein was intramolecularly cross-linked with glutaraldehyde. The GraFix method was only applied for the most promising Gcd of *C. symbiosum*. In addition, the chemically fixed protein remained stable, even when the NaCl concentration was reduced during SEC from 400 mM to 100–200 mM (Figure 1B). This finding was very important since meaningful LILBID-MS and EM studies can only be performed with salt concentrations below 200 mM. The reduction of the salt concentration without chemical fixation resulted in aggregated and dissociated Gcd complexes.

For further cryo-EM studies the purified Gcd complex had to be concentrated. However, the applications of Amicon centrifugal concentrators (Millipore) or Centrisart reverse centrifugal concentrators (100-kDa cut off membrane) failed, because 95% of the protein complex dissociated into the head and membrane components even after chemical fixation as seen in SEC profiles (Supplementary Figure S1F) and negative stain EM images (Supplementary Figure S1G). One step forward was to solubilize larger amounts of the Gcd complex and to use smaller amounts of monomeric avidin agarose to elute the protein at a high concentration from the column. The concentration of the obtained Gcd complex was 0.5–1 mg/ml.

### Molecular Mass Determination of Gcd From *C. symbiosum*

**FIGURE 2:**
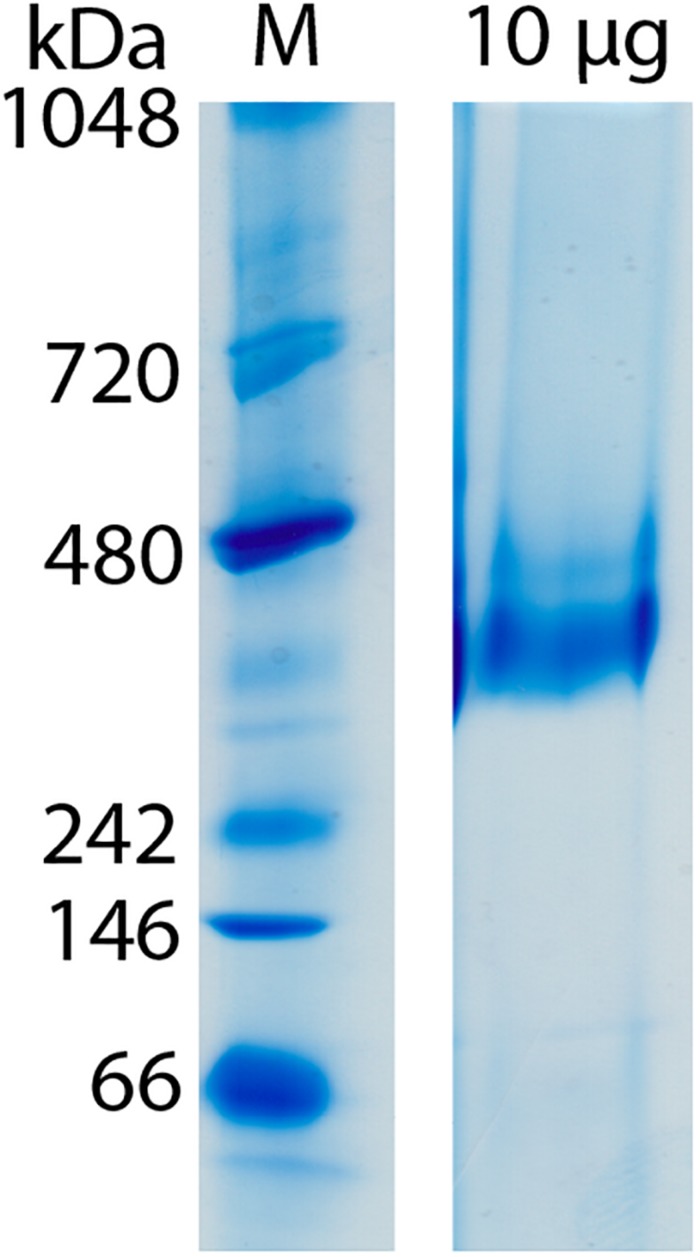
Molecular mass determination of Gcd by clear-native PAGE. The gel was performed directly after monomeric avidin agarose affinity chromatography. Unfixated and unconcentrated Gcd was present in buffer A.

Clear-native PAGE analysis of chemically unfixed and unconcentrated Gcd-detergent complexes (10 μg) in buffer A showed a pronounced protein band at 380 ± 20 kDa (Figure 2). No striking aggregates were detected with this method. Molecular mass analysis with SEC essentially revealed a very broad peak with a maximum of ca. 450 kDa for the chemically fixed Gcd complex (Figure 1B) reflecting the variably sized detergent belt. Several SEC-multi angle light scattering experiments on chemically fixed Gcd complexes (50 nM enzyme in buffer A, 17°C) resulted in the same retention times, but the scattering intensity and the calculated molecular mass varied considerably. Notably, the polydisperse index *M*_*w*_ (weight-averaged mass)/*M*_*n*_ (number averaged mass) was always 1.0 indicating a high monodispersity of the sample (Slotboom et al., 2008).

**FIGURE 3:**
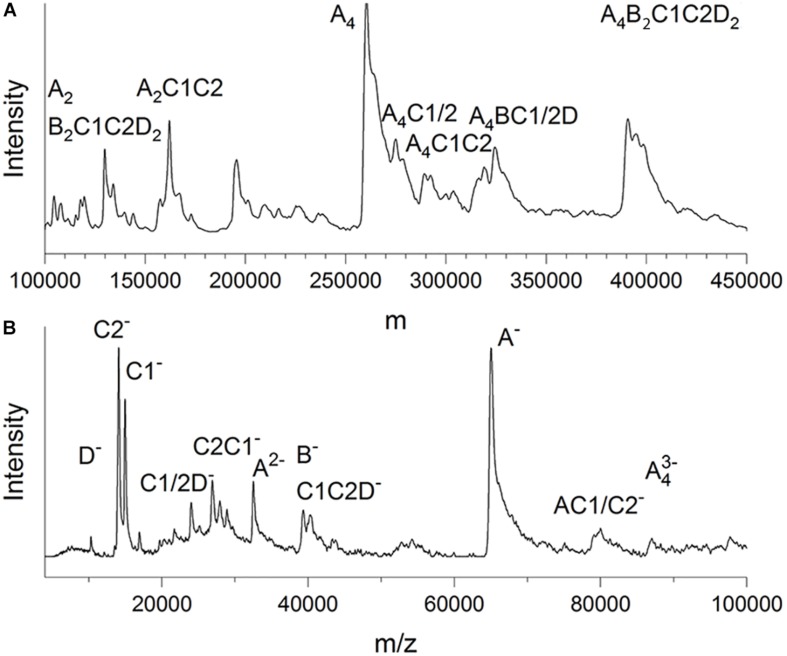
LILBID-MS. **(A)** Deconvoluted zero-charge mass spectrum (Marty et al., 2015) of Gcd in soft detection mode (14 mJ laser power). The deconvolution calculates a zero-charge spectrum by relating the intensities of multiple differently charged ions of the same species to the non-charged state. **(B)** Mass spectrum of Gcd in harsh detection mode (23 mJ laser power). The sample is dissociated into smaller fragments by the laser energy. Only peaks with biologically plausible subunit compositions were marked.

LILBID-MS data were recorded in soft and harsh detection modes by using two different intensities of a mid-IR laser (Figure 3). The protein droplets contained 3–7 μM glutaraldehyde-fixed and unconcentrated Gcd complexes in 50 mM Tris–HCl, pH 7.0, 0.05% DDM and 200 mM NaCl. In the soft detection mode (14 mJ laser power) signals corresponding to a maximum molecular mass of ca. 390 kDa were detectable. This value is close to that determined from the clear native PAGE gel and proposes a subunit composition of 4 GcdA, 2 GcdB, GcdC1, GcdC2, and 2 GcdD (Figure 3A). For comparison, the DNA-deduced mass is 379 kDa. Furthermore, peaks at *m*/*z* of 260 and 130 kDa could be reliably assigned as a GcdA tetramer and GcdA dimer (Supplementary Figure S2).

Under harsh laser pulse conditions (23 mJ) a series of spectra with 26 μM chemically unfixed Gcd complexes yielded peaks for several subcomplexes and the five subunits GcdA, GcdB, GcdC1, GcdC2, and GcdD at 65, 35, 15, 14, and 10 kDa, respectively (Figure 3B), which confirmed the results of mass fingerprinting. Peaks representing further subcomplexes were tentatively interpreted (Figure 3B). LILBID MS experiments were also performed with Gcd which was not cross-linked. Under soft laser pulse conditions, a series of spectra yielded also peaks for several subcomplexes and the five subunits, respectively (Supplementary Figure S2). The suspicion that the intact Gcd complex was absent, was corroborated by measuring LILBID-MS data by a stepwise ionization of the sample over a period of about 25 min resulting in decreased initial peaks higher than 50 kDa and increased peaks less than 50 kDa (Supplementary Figure S2). Altogether, the LILBID-MS method is also a powerful analytical method for investigating the integrity of fragile protein complexes.

### Negative-Stain EM of Gcd From *C. symbiosum*

**FIGURE 4:**
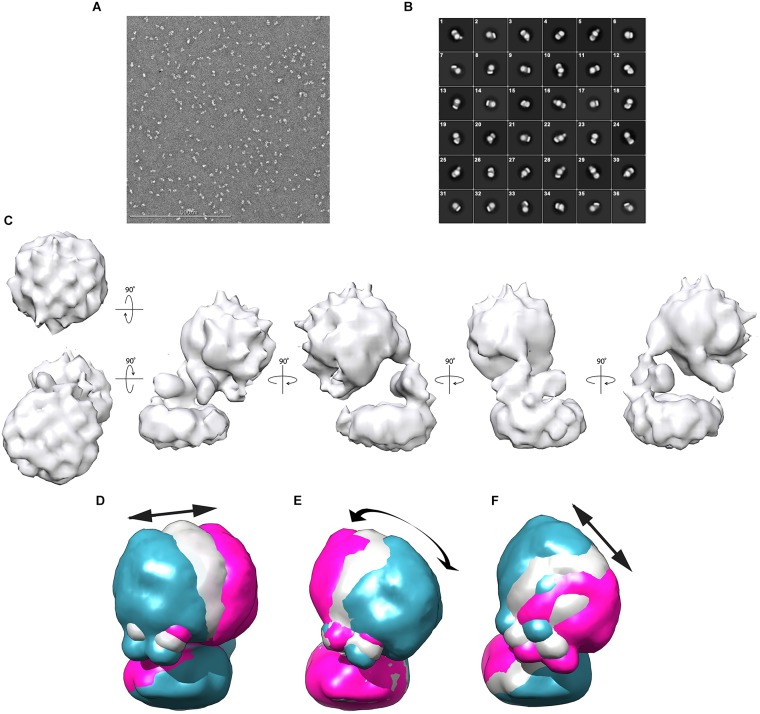
Negative-stain EM analysis from *C. symbiosum* Gcd. **(A)** Electron micrograph of Gcd stained with uranyl formate. **(B)** 2D classification of Gcd particles. **(C)** Low-resolution map of Gcd viewed from different orientations. **(D–F)** Conformational flexibility of the Gcd complex illustrated by the three directions of motion with the largest amplitude of the hydrophilic head (Supplementary Figure S3). The three membrane bases were superimposed. The blue and pink models show the maximum amplitudes of head; the gray model represents an average value of the motion direction. The arrows illustrate the direction of the movements and the approximate maximum amplitude.

For negative-stain EM analysis, glutaraldehyde-fixed Gcd was applied and adjusted at a salt concentration of 200 mM NaCl. The sample (50–80 nM) was loaded onto freshly glow-discharged carbon-coated copper grids and stained with 2% (w/v) uranyl formate. The Gcd complex sample was mostly visible as intact and separated particle with only minor amounts of aggregates and smaller dissociation products (Figure 4A).

112,399 negative-stained particles were extracted at a pixel size of 5.36 Å in a 56 × 56-pixel box and subjected to 2D classification. Visual selection of class averages with interpretable features resulted in 36 well-defined classes of 36,905 particles (Figure 4B). The particles of the selected classes were re-centered and re-extracted at full pixel size of 2.68 Å. Particles belonging to those 2D classes of highest quality were averaged and a reconstructed 3D density map was calculated (Figure 4C).

**FIGURE 5:**
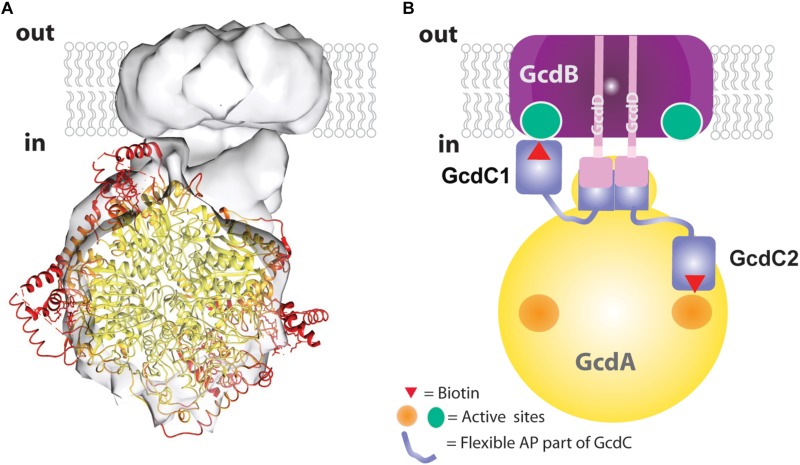
Structural and mechanistic interpretation of the Gcd reconstruction map. **(A)** The structure of tetrameric GcdA (pdb 1PIX) is modeled into the map thereby drawing the coordinates with B-factors <30 Å^2^ in yellow and B-factors >50 Å^2^ in red. **(B)** Subunit assignment. The GcdB dimer (purple) and the N-terminal helix of both GcdDs (pink) form the membrane region. A short AP-rich stretch (light-pink) joins the N-terminal helix and the C-terminal region (pink) of GcdD. The C-terminal region of the two GcdDs constitute together with a stretch of GcdA (yellow) and the N-terminal region of GcdC1/C2 (blue) the linker between the globular parts. The C-terminal regions of GcdC1/C2 (blue) covalently bind biotin. Both regions are linked by flexible AP-rich regions as indicated by the thin light-blue lines. The flexible region allows the biotin bound to the C-terminal region to interact with the active sites of GcdA (right side) and GcdB (left side) within the catalytic cycle.

Accordingly, the 3D density map of the Gcd complex is composed of two globular parts. The smaller cylindric base is ca. 85 Å in diameter and 35 Å high and is primarily assigned to the membrane spanning GcdB dimer (Figure 4C). The larger globular head has a diameter of 80–90 Å. It approximately fits to the size of a GcdA tetramer (Figure 5A). The hydrophilic head and the membrane-spanning base, whose profiles are compatible with the expected two-fold symmetries, are connected by the remaining density which is weaker and therefore less defined. Its composition is unclear and might include segments of GcdA, GcdC1, GcdC2, and GcdD (see below).

Since the 2D classifications indicate variable heterogeneous motions of the Gcd particles, multi-body refinement in RELION (Scheres, 2012) was used to visualize the structural heterogeneity of the Gcd complex. For calculations, the clearly visible membrane base and the solvent-exposed head were used as independently moving rigid bodies. Their relative orientations were analyzed over all particles of the images and the three motions with the largest displacements are shown in Figures 4D–F. Further directions of movement, specified as eigenvectors, were given in the Supplementary Figure S3A. The amplitudes extend up to nearly 50 Å in some of the particles thereby documenting the high conformational flexibility of the solvent-exposed head relative to the membrane base (Supplementary Figures S3B–D).

## Discussion

Biotin-dependent Na^+^ translocating decarboxylases play a key role in fermenting bacteria metabolizing β-keto acids and their thioester analogs by using an exergonic decarboxylase reaction and not a redox reaction as usually found in ion-gradient forming processes of energy conservation. For structural and mechanistic studies, a pure, rather homogeneous and complete protein complex is a prerequisite. Therefore, after diverse screening and optimization attempts, we succeeded in working out a purification protocol for the Gcd complex from *C. symbiosum*. Key factors in handling the fragile protein complex are cross-linking with glutaraldehyde and omission of concentration procedures. Native PAGE and SEC experiments resulted in masses of 380 ± 30 kDa and 450 ± 80 kDa (Figures 1, 2). The less shape and detergent based LILBID-MS data resulted in an approximate mass of 390 kDa. Derived from a 3D map of negative-stain EM data (Figure 4), Gcd occupies a volume of 5.4 × 10^5^ Å^3^ (Chimera, recommended contour level: 9) (Pettersen et al., 2004). Based on the equation *V*(Å^3^) = 1.212 Å^3^ (Da)^–1^ × molecular mass (Da) (Erickson, 2009), the approximate mass is 445 kDa. Taking the errors of each method into account and different detergent contents of the samples, the values match adequately. The measured molecular masses are most compatible with a subunit composition of 4 GcdA, 2 GcdB, GcdC1, GcdC2, and 2 GcdD. A tetrameric GcdA was also reported for the X-ray structures of the *A. fermentans* and *C. symbiosum* enzymes (Wendt et al., 2003; Kress et al., 2009). This unusual stoichiometric relationship has precursors although one would expect that a tetrameric GcdA should assemble with 4 copies of GcdB, GcdC, and GcdD resulting in a molecular mass of 498 kDa, about 125% higher than the measured one. An example of a 4:2:2 relationship is found in the electron transfer protein-propionyl-CoA dehydrogenase complex (Etf-Pcd) from *Clostridium propionicum*, which is composed of 4 Pcd, 2 EtfA, and 2 EtfB subunits (Hetzel et al., 2003). The crystal structure of the closely related Etf-butyryl-CoA dehydrogenase (Etf-Bcd) complex from *Clostridium difficile* revealed that one EtfAB monomer binds to two Bcd subunits (Demmer et al., 2017).

The EM map was interpreted in a fashion that the larger sphere-shaped solvent-exposed head roughly corresponds to the tetrameric GcdA and the smaller cylindric membrane-spanning base to the GcdB dimer. The borders of the globular parts are approximately 25 Å apart and bridged by a linker. By integrating primary structure data, a more differentiated picture of the model can be proposed which resembles a 20-years old cartoon of Gcd from *A. fermentans* (Braune et al., 1999). The N-terminal membrane-spanning α-helix of both GcdDs serve most likely as membrane anchor associated with the GcdB dimer. The C-terminal cytoplasmic region of both GcdDs constitutes the linker to GcdA as observed in Oad (Dahinden et al., 2005) together with the N-terminal region of GcdC1/C2 (Figure 5B). This assumption is plausible as the N-terminal regions of GcdC1/C2 probably act as an anchor for the moving C-terminal region optimally positioned between GcdA and GcdB. According to former hypotheses, the C-terminal region of GcdC1/C2 bind biotin and swings via a flexible AP-rich connector as N^1^-carboxybiotin from GcdA to GdcB and back after the decarboxylation of biotin (Eqs 1 and 2; Figure 5B). Since Gcd was analyzed without its substrates glutaconyl-CoA or crotonyl-CoA, the biotin bound to GcdC1/C2 is probably attached to the active sites of the carboxy transferase reactions on GcdA. The here described model of Gcd is somewhat different from the symmetric and asymmetric models derived from crystal structures of GcdA, because they do not consider GcdD and the space of about 25 Å between the GcdA and GcdB (Wendt et al., 2003; Kress et al., 2009).

Negative-stain EM analysis indicates different orientations of the cytoplasmic head relative to the membrane base (Figures 4D–F). This conformational variety appears to be due to the high flexibility of the linker which is visible in its weak EM density compared to that of the globular parts. The AP-rich region between the membrane-spanning and cytoplasmic part of GcdD might cause the flexibility of the linker and thus the large conformational variety of the head (Figures 4D–F). Information at higher resolution is necessary to understand the relationship between the flexibility of the linker and the catalytic cycle between the two active sites.

## Data Availability Statement

The negative stain EM map was deposited at the wwPDB under accession number EM-10743.

## Author Contributions

SV, UE, and WB designed the research project. SV performed or significantly contributed to all experiments and interpreted most data. SP conducted the EM experiments, NH and NM the LILBID-MS experiments. SV, UE, and WB wrote the manuscript with contributions of all authors.

## Conflict of Interest

The authors declare that the research was conducted in the absence of any commercial or financial relationships that could be construed as a potential conflict of interest.
