# Supplementary material for: Molecular and Low-Resolution Structural Characterization of the Na+-Translocating Glutaconyl-CoA Decarboxylase From Clostridium symbiosum
Source: Front Microbiol. 2020 Mar 31;11:480. doi: 10.3389/fmicb.2020.00480 (PMC7145394; doi:10.3389/fmicb.2020.00480)
Supplement: Supplementary file 1 [file Data_Sheet_1.docx]

**SUPPLEMENTARY MATERIAL**

**Molecular and low-resolution structural characterization of a Na^+^-translocating glutaconyl-CoA decarboxylase from *Clostridium symbiosum***

**Stella Vitt^1,2*^, Simone Prinz^3^, Nils Hellwig^4^, Nina Morgner^4^, Ulrich Ermler^1^, Wolfgang Buckel^2*^**

*^1^ Department of Molecular Membrane Biology, Max Planck Institute of Biophysics, Max-von-Laue-Str. 3, 60438 Frankfurt am Main, Germany,^2^ Philipps-Universität Marburg, Faculty of Biology, Karl-von-Frisch Straße 8, 35043 Marburg, Germany, ^3^Department of Structural Biology, Max Planck Institute of Biophysics, Max-von-Laue-Str. 3, 60438 Frankfurt am Main, Germany, ^4^ Institute of Physical and Theoretical Chemistry, Goethe University Frankfurt, Max-von-Laue-Str. 7, 60438 Frankfurt am Main, Germany*

***Correspondence:** Wolfgang Buckel

Buckel@biologie.uni-marburg.de

Stella Vitt

Stella.vitt@mpibp-frankfurt.mpg.de

**
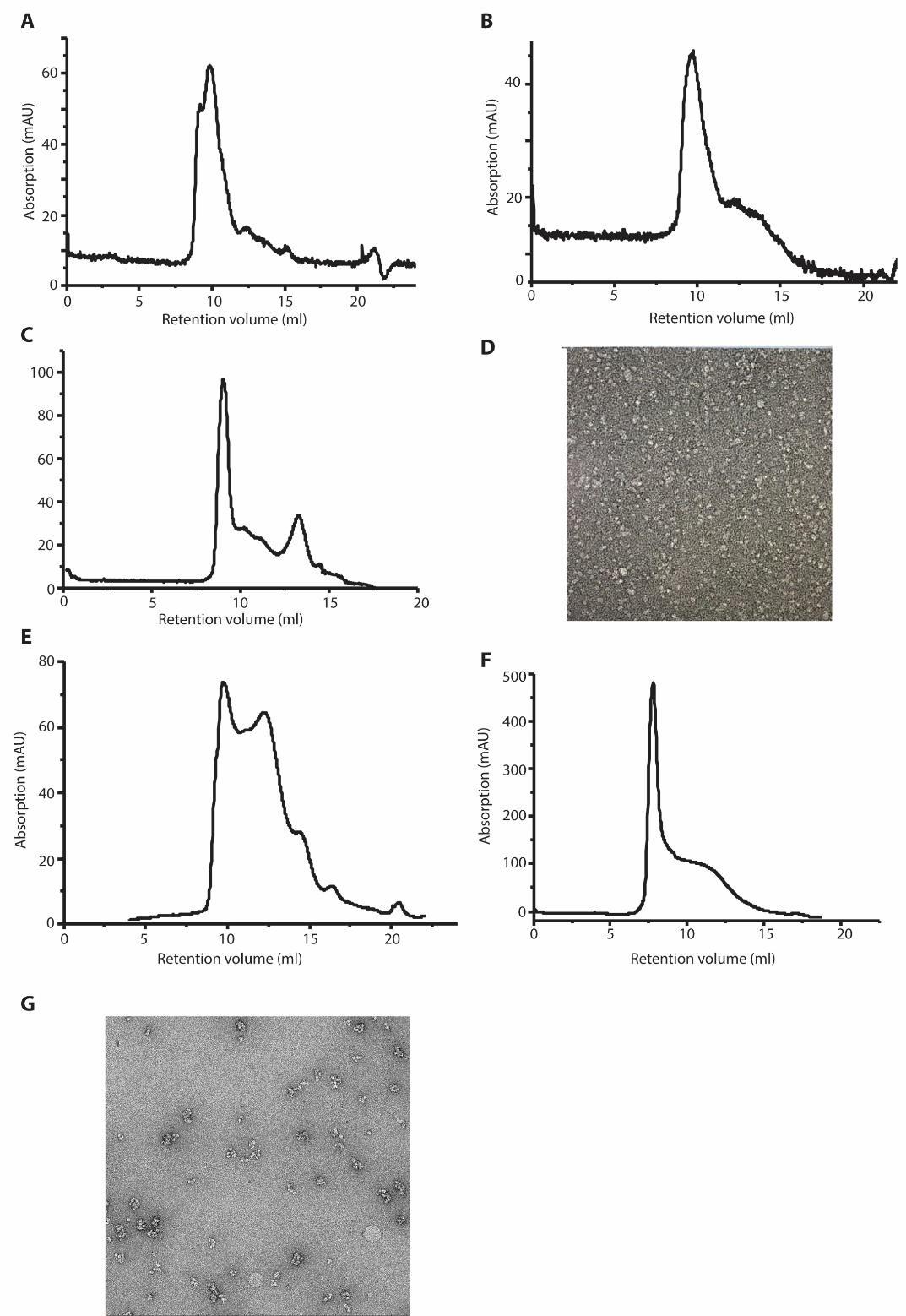
**

**FIGURE S1 |** Quality analysis of the Gcd complex. Gel filtration profiles of Gcd from *F. nucleatum* **(A)**, *P. asaccharolyticus* **(B)** and *A. fermentans* (**C**). The negative stain EM image of Gcd from *A. fermentans* **(D)** showing a highly degraded, heterogeneous protein complex**.** Gel filtration profile of Gcd of *C. symbiosum* before and after concentration **(E, F)** and its EM image after chemical fixation and concentration **(G)**. A single Gcd complex elutes at a retention volume of 13 ml while the peak at 8-12 ml retention volume is attributed to aggregated protein complex. The peak at 10 ml retention volume demonstrates the aggregated state of the *F. nucleatum* **(A)** and *P. asaccharolyticus* **(B)** Gcd. The gelfiltration profile and EM images of C. symbiosum Gcd (**F, G**) indicates that the protein complex is largely aggregated after concentration.


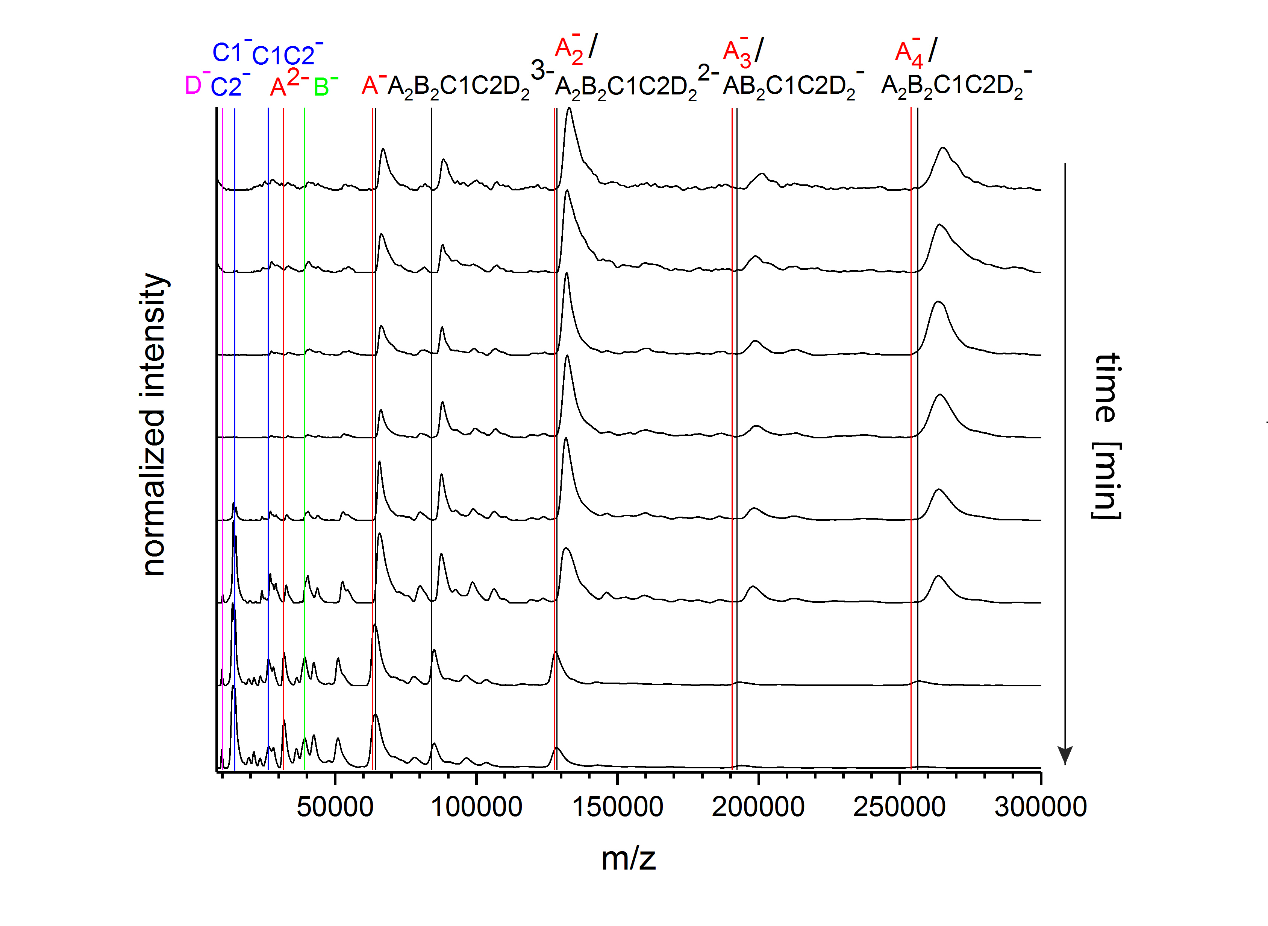
**FIGURE S2 |** Time-dependent LILBID-MS spectra of chemically unfixated Gcd under soft conditions. Under soft laser pulse condition a series of spectra with 26 μM chemically unfixated Gcd yielded peaks for several subcomplexes and the five subunits GcdA, GcdB, GcdC1, C2 and GcdD, respectively, which confirmed the results of mass fingerprinting. The concentration of charged ions of the complex and the peaks less than 50 kDa increased. In the measured period of about 25 minutes NaCl diffuses out of the Gcd complex. Its mass decreases, which explained the reducing m/z values over time.


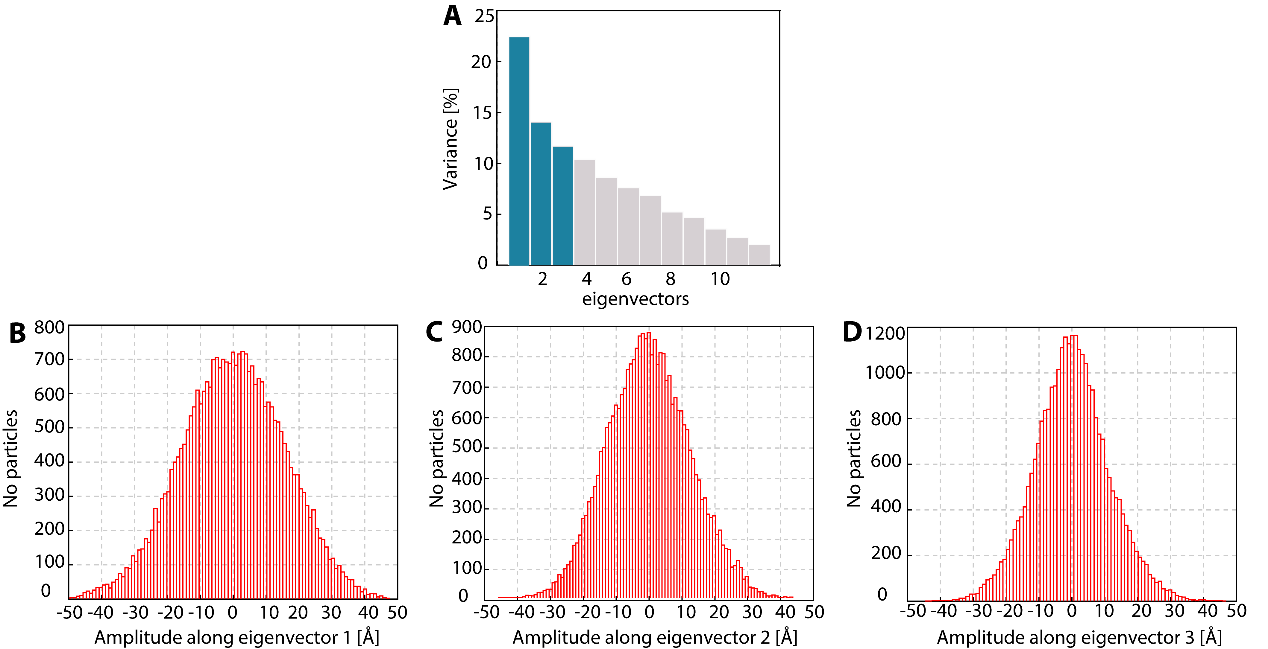


**FIGURE S3 |** Conformational flexibility of the Gcd complex. **(A)** Directions of movement (represented by eigenvectors) of the cytosolic head part relative to the membrane part on the basis of all intact particles of the dataset. Maps are shown in **Figure 4 D-F** based on the eigenvectors 1-3 (petrol) representing the three direction with the largest displacements. The variance is a measure for the displacement. (**B–D)** Histogram of the number of particles with the same amplitudes of the head groups shown for the directions 1, 2 and 3.
